# Workplace standing time and the incidence of obesity and type 2 diabetes: a longitudinal study in adults

**DOI:** 10.1186/s12889-015-1353-x

**Published:** 2015-02-10

**Authors:** Jean-Philippe Chaput, Travis J Saunders, Mark S Tremblay, Peter T Katzmarzyk, Angelo Tremblay, Claude Bouchard

**Affiliations:** Healthy Active Living and Obesity Research Group, Children’s Hospital of Eastern Ontario Research Institute, 401 Smyth Road, Ottawa, ON K1H 8L1 Canada; Department of Applied Human Sciences, Faculty of Science, University of Prince Edward Island, 550 University Ave, Charlottetown, PE C1A 4P3 Canada; Physical Activity and Obesity Epidemiology Laboratory, Pennington Biomedical Research Center, 6400 Perkins Road, Baton Rouge, LA 70808-4124 USA; Department of Kinesiology, Faculty of Medicine, 2300 de la Terrasse, Laval University, Quebec City, QC G1V 0A6 Canada; Human Genomics Laboratory, Pennington Biomedical Research Center, 6400 Perkins Road, Baton Rouge, LA 70808-4124 USA

**Keywords:** Standing, Sitting, Obesity, Diabetes, Longitudinal study, Adults

## Abstract

**Background:**

It is increasingly recognized that standing represents a simple solution to extended periods of sitting. However, it is currently unknown whether workplace standing time is prospectively associated with a lower incidence of chronic diseases. The objective of this study was to examine the association between workplace standing time and the incidence of overweight/obesity (OW/OB) and impaired glucose tolerance/type 2 diabetes (IGT/T2D) in adults.

**Methods:**

A longitudinal analysis from the Quebec Family Study (Canada) was conducted on 293 participants, aged 18 to 65 years, followed for a mean of 6 years. Information on self-reported occupational standing time as well as several covariates was collected at both baseline and follow-up. Outcome measures included the development of OW/OB (*i.e.* body mass index ≥25 kg/m^2^) and IGT/T2D (*i.e.* 2-h postload plasma glucose level ≥7.8 mmol/L).

**Results:**

The incidence rates of OW/OB and IGT/T2D over the 6-year follow-up period were 17.4% and 12.6%, respectively. Significant negative associations were observed between the amount of occupational standing time and the development of outcome measures. However, the associations were no longer significant after adjustment for age, sex, smoking habits, total annual family income, daily caloric intake, and submaximal working capacity. In age- and sex-adjusted logistic regression analysis, significant negative linear trends were observed across levels of standing time and the outcome variables. However, the associations were no longer significant after further adjustment for the other covariates. Finally, we observed that the change in standing time from baseline to year 6 was significantly associated with the development of outcome measures, with higher incidence rates in adults reporting a reduction in standing time at follow-up. However, the associations became non-significant after adjustment for covariates.

**Conclusions:**

Greater occupational standing time is not sufficient in and of itself to prevent the development of OW/OB and IGT/T2D in adults. Future efforts are needed to better understand the potential benefits of higher amounts of standing time throughout the day on the prevention of chronic diseases.

## Background

Accumulating evidence associates sedentary behavior (*e.g.* sitting) with adverse health outcomes including obesity and type 2 diabetes [1-5]. Studies also suggest that the effects of sedentary behavior on health indicators may be independent of moderate-to-vigorous physical activity [6-8]. However, many low-energy-expenditure activities can be classified as “sedentary behaviors” (*e.g.* reading, television viewing, driving) with effects that may be different on health indicators depending on the specific behavior [9-12]. Among behaviors at the low end of the energy expenditure continuum, standing has not received much attention in its ability to prevent the development of adverse health outcomes.

Breaking up sedentary time has recently been shown to be promising for improving cardiometabolic health [13-18]. One of the easiest ways to interrupt prolonged sitting is to stand up. Although standing quietly involves low levels of energy expenditure (approximately 1.2 METs), it engages a large muscle mass in the lower extremities and may represent a healthier alternative to sitting. Interestingly, a recent study reported that greater time spent standing was associated with a lower risk of mortality in adults [19]. Small-scale intervention studies also suggest that replacing workplace sitting with standing may reduce the glycemic response to a test meal [20,21]. However, it is unknown whether workplace standing time is prospectively related to a lower incidence of chronic diseases. Given that standing represents a simple solution to reduce extended periods of sitting, it is of interest to understand the association between standing and cardiometabolic health.

Therefore, the objective of this study was to examine the association between occupational standing time and the incidence of overweight/obesity (OW/OB) and impaired glucose tolerance/type 2 diabetes (IGT/T2D) in adults. We hypothesized that greater amounts of standing would be related to a lower incidence of the above-mentioned outcome measures.

## Methods

### Participants

The Quebec Family Study was initiated at Laval University in 1978. The primary objective of this study was to investigate the genetics of fitness, body composition and cardiovascular risk factors. In phase 1 of the study (1979 to 1982), a total of 1650 individuals from 375 families (nuclear families with biological or adopted offspring, pairs of twins of both types, and uncle/aunt and nephew and niece when available) were recruited and assessed. Recruitment was conducted irrespective of body weight during phase 1, resulting in a cohort with a wide range of body mass index levels. In phase 2 (1989–1997) and 3 (1998–2002), 100 families from phase 1 were retested, and an additional 123 families with at least 1 parent and 1 offspring with a body mass index of 32 kg/m^2^ or higher were added to the cohort. Families were all of French descent and were living for the most part within 80 km of Quebec City (Canada). Details on recruitment procedures and other aspects of the Quebec Family Study can be found elsewhere [22,23]. This cohort thus represents a mixture of random sampling and ascertainment through obese individuals. The present analyses are based on participants tested in phases 2 and 3 because some measurements were not available in phase 1. Adults between 18 and 65 years of age were selected for longitudinal analyses (*n* = 293). A total of 23 participants were excluded because they were outside this age range. The mean duration of follow-up between phase 2 and 3 was 6.0 ± 0.9 years. All subjects provided written informed consent to participate in the study. The project was approved by the Medical Ethics Committee of Laval University.

### Standing time assessment

To assess the primary exposure variable, participants completed a questionnaire and answered the following question: “How much time do you spend standing during your main occupation (*e.g.* at work)?” Responses included (*i*) all the time, (*ii*) most of the time, (*iii*) half of the time, and (*iv*) rarely/never. The assessment was performed at both baseline and after 6 years.

### Assessment of outcome variables

#### Overweight/obesity (OW/OB)

At both baseline and year 6, height was measured to the nearest 0.1 cm using a stadiometer, and body weight was measured to the nearest 0.1 kg using a digital panel indicator scale (Beckman Industrial Ltd, Model 610/612, Scotland, UK). Body mass index (BMI) was calculated as body weight divided by height squared (kg/m^2^). OW/OB was defined as a BMI ≥25 kg/m^2^, in agreement with well-established international standards [24]. OW/OB were combined in the analyses due to the low incidence of OB alone.

#### Impaired glucose tolerance/type 2 diabetes (IGT/T2D)

A 75 g oral glucose tolerance test was performed in the morning after a 12-h overnight fast. Blood samples were collected in tubes containing EDTA and Trasylol (Miles Pharmaceutics, Rexdale, ON, Canada) through a venous catheter from an antecubital vein at −15, 0, 15, 30, 45, 60, 90, 120, 150 and 180 min. Plasma glucose concentration was measured enzymatically [25], and fasting glucose concentration was calculated as the mean of the −15 and 0 min concentrations. T2D and IGT were defined according to the American Diabetes Association and the World Health Organization criteria [26,27]. T2D was defined as use of insulin or a hypoglycemic agent, a fasting plasma glucose level of 126 mg/dL or more (≥7.0 mmol/L), or a 2-h postload plasma glucose level of 200 mg/dL or more (≥11.1 mmol/L). On the other hand, IGT was defined as a 2-h postload plasma glucose level of 140 mg/dL or more (≥7.8 mmol/L) in participants not meeting the criteria for T2D. The measurements were performed the same way at both baseline and follow-up. Similar to OW/OB, we combined IGT and T2D cases.

#### Covariates

Numerous variables were measured via self-reported questionnaires at baseline and year 6. These included age, sex, smoking habits (smoker or nonsmoker), and total annual family income (Canadian dollars per year). Additionally, daily energy intake (kcal/day) was assessed with a 3-day food record (two week days and one weekend day). This method of dietary assessment has been shown to provide a reasonably reliable measurement of food intake in this population [28]. Finally, physical work capacity at a heart rate of 150 bpm (PWC_150_), determined by a progressive exercise test on a modified Monark cycle ergometer, was used as an indicator of cardiorespiratory fitness, as previously described [29]. PWC_150_ is expressed in kilopond per minute per kilogram (kpm/kg) to take individual differences in body weight into account. These 6 covariates were chosen because of their association with the exposure and outcomes and on the basis of previous research [1,2,17,19]. Given that over-fitting can be a concern with our relatively small sample size, different analyses were conducted to assess model fit (including testing for multicollinearity) and results demonstrated that our models had good predictive performance and were not subject to over-fitting.

#### Statistical analysis

To determine if men and women could be combined, sex-by-standing time interactions were assessed for all dependent variables. No significant interactions were found, therefore data for both sexes were combined to maximize power. Baseline characteristics of participants by standing time category were compared by analysis of variance (continuous variables) or chi-squared test (categorical variables). Among participants free of the outcome of interest at baseline (*n* = 151 normal weight and *n* = 260 without IGT/T2D), the incidence of OW/OB and IGT/T2D by standing time category over the 6-year follow-up period was calculated and a chi-squared test was used to assess statistical significance. Multivariable logistic regression analysis was also used to evaluate the risk for the development of OW/OB and IGT/T2D according to the amount of standing time. The category “rarely/never” was used as the reference group. The results from two models are presented: (*i*) adjusted for age and sex and (*ii*) additionally adjusted for smoking habits, total annual family income, daily caloric intake, and submaximal working capacity. Odds ratios (OR) and 95% confidence intervals (CI) were reported. Finally, a chi-squared test was used to compare the incidence of OW/OB and IGT/T2D across categories of changes in standing time between baseline and year 6 (decreased, maintained, increased). Because some individuals in this study came from the same nuclear family and are biologically related, we adjusted for clustering in the analyses using the generalized estimating equations statistical method to avoid underestimation of standard deviations. This procedure allowed us to model standing time and covariates as repeated measures at two time points (baseline and 6 years later), thus taking into account both measures over time. A 2-tailed *P* value of less than 0.05 was the threshold to indicate statistical significance. All statistical analyses were performed using JMP version 11 (SAS Institute, Cary, NC).

## Results

Baseline characteristics of participants within each standing time group are shown in Table 1. Among the 293 Caucasian participants of this study, 33% reported standing rarely or never, 23% half of the time, 19% most of the time, and 25% all the time during their main occupation.

**Table 1:** **Baseline descriptive characteristics of participants across levels of standing time**

	**Rarely/Never**	**Half of the time**	**Most of the time**	**All the time**	***P***
	**(** ***n*** **= 97)**	**(** ***n*** **= 66)**	**(** ***n*** **= 57)**	**(** ***n*** **= 73)**	
Age (years)	37.3 ± 14.7	42.2 ± 13.7	42.6 ± 14.0	35.6 ± 12.2	<0.01
Sex					
Men (%)	56	37	37	43	
Women (%)	44	63	63	57	0.04
Body mass index (kg/m^2^)	25.7 ± 5.5	26.3 ± 6.3	25.1 ± 4.5	25.2 ± 5.6	0.61
Waist circumference (cm)	85.0 ± 14.9	84.4 ± 16.8	82.2 ± 12.6	81.9 ± 15.6	0.53
Weight status^a^					
Normal weight (%)	50	52	58	59	
Overweight (%)	35	22	29	26	
Obese (%)	15	26	13	15	0.35
Diabetes status^b^					
Normal (%)	84	76	81	95	
IGT (%)	10	20	14	3	
Type 2 diabetes (%)	6	4	5	2	0.08
Smoking habits					
Nonsmoker (%)	84	87	85	79	
Smoker (%)	16	13	15	21	0.64
Total annual family income ($C)	66,848 ± 26,846	54,180 ± 24,886	52,700 ± 21,740	56,323 ± 23,238	<0.01
Energy intake (kcal/day)	2,329 ± 634	2,211 ± 648	2,226 ± 647	2,498 ± 979	0.11
PWC_150_ (kpm/kg)	9.3 ± 2.9	8.9 ± 2.4	8.5 ± 3.6	9.1 ± 3.2	0.68

Values are presented as mean ± SD for continuous variables and percentage (%) for categorical variables. Statistical significance was assessed by analysis of variance with continuous variables and by a chi-squared test with categorical variables. ^a^Normal weight (body mass index between 18.5 and 24.9 kg/m^2^), overweight (body mass index between 25.0 and 29.9 kg/m^2^) and obese (body mass index ≥30 kg/m^2^). ^b^Type 2 diabetes and impaired glucose tolerance were defined according to the American Diabetes Association and the World Health Organization criteria [26,27]. *Abbreviations: IGT* impaired glucose tolerance; *PWC*_*150*_ physical work capacity at a heart rate of 150 bpm.

**Figure 1:**
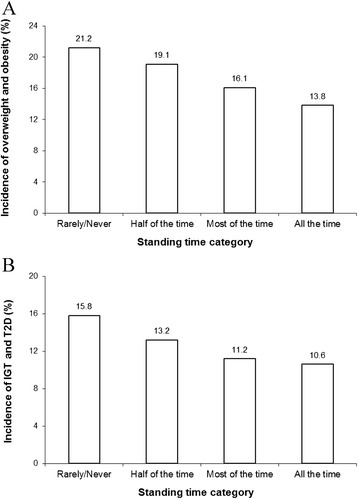
**Development of overweight/obesity (A) and impaired glucose tolerance/type 2 diabetes (IGT/T2D) (B) according to categories of standing time over the 6-year follow-up period in adults.** Data are presented as percentage. Overweight/obesity was defined as a body mass index ≥25 kg/m^2^. Impaired glucose tolerance/type 2 diabetes was defined according to the American Diabetes Association and the World Health Organization criteria [26,27]. Statistical significance was assessed by a chi-squared test (*P* < 0.05 for all analyses). Standing categories: rarely/never (*n* = 97), half of the time (*n* = 66), most of the time (*n* = 57) and all the time (*n* = 73).

Over the 6-year follow-up period, 51 new cases of OW/OB (17.4%) and 37 new cases of IGT/T2D (12.6%) were observed (there were 151 normal weight participants and 260 without IGT/T2D at baseline). As shown in Figure 1, a negative dose–response association was observed between the amount of standing time and the development of OW/OB and IGT/T2D (*P* < 0.05). However, the associations were no longer significant after adjusting for covariates (age, sex, smoking habits, total annual family income, daily caloric intake, and submaximal working capacity) (data not shown). Specifically, total annual family income and submaximal working capacity were the main confounders of the relationship (data not shown).

The results of the multivariable logistic regression analysis assessing the relationship between standing time and the development of OW/OB and IGT/T2D are presented in Table 2. In the age- and sex-adjusted analyses, there were significant negative linear trends across levels of standing time and the outcome variables. However, the associations were no longer significant after further adjustment for smoking habits, total annual family income, daily caloric intake and submaximal working capacity. Here again, the addition of total annual family income and submaximal working capacity to the models resulted to the greater attenuation of ORs (data not shown). Of note, the delta ORs between the “Rarely/Never” standing group and the “All the time” standing group were reduced from a difference of 33% (0.94 to 0.63) to 12% (0.97 to 0.85) and from a difference of 27% (0.91 to 0.66) to 5% (0.96 to 0.91) for OW/OB and IGT/T2D, respectively, after multivariable adjustment.

**Table 2:** **Risk for the incidence of overweight/obesity and impaired glucose tolerance/type 2 diabetes by standing time category over the 6-year follow-up period**

	**Rarely/Never**	**Half of the time**	**Most of the time**	**All the time**	***P *** **for trend**
	**(** ***n*** **= 97)**	**(** ***n*** **= 66)**	**(** ***n*** **= 57)**	**(** ***n*** **= 73)**	
Overweight/obesity					
Age- and sex-adjusted OR (95% CI)	1.00	0.94 (0.53-1.48)	0.79 (0.34-1.44)	0.63 (0.21-1.22)	<0.05
Multivariable-adjusted OR^a^ (95% CI)	1.00	0.97 (0.55-1.51)	0.88 (0.42-1.57)	0.85 (0.31-1.37)	0.19
Impaired glucose tolerance/type 2 diabetes					
Age- and sex-adjusted OR (95% CI)	1.00	0.91 (0.47-1.49)	0.80 (0.31-1.53)	0.66 (0.22-1.27)	<0.05
Multivariable-adjusted OR^a^ (95% CI)	1.00	0.96 (0.51-1.53)	0.90 (0.40-1.65)	0.91 (0.35-1.49)	0.36

^a^Adjusted for age, sex, smoking habits, total annual family income, daily caloric intake, and submaximal working capacity. Overweight/obesity was defined as a body mass index ≥25 kg/m^2^. Impaired glucose tolerance/type 2 diabetes was defined according to the American Diabetes Association and the World Health Organization criteria [26,27]. *Abbreviations: OR* odds ratio, *CI* confidence interval.

**Figure 2:**
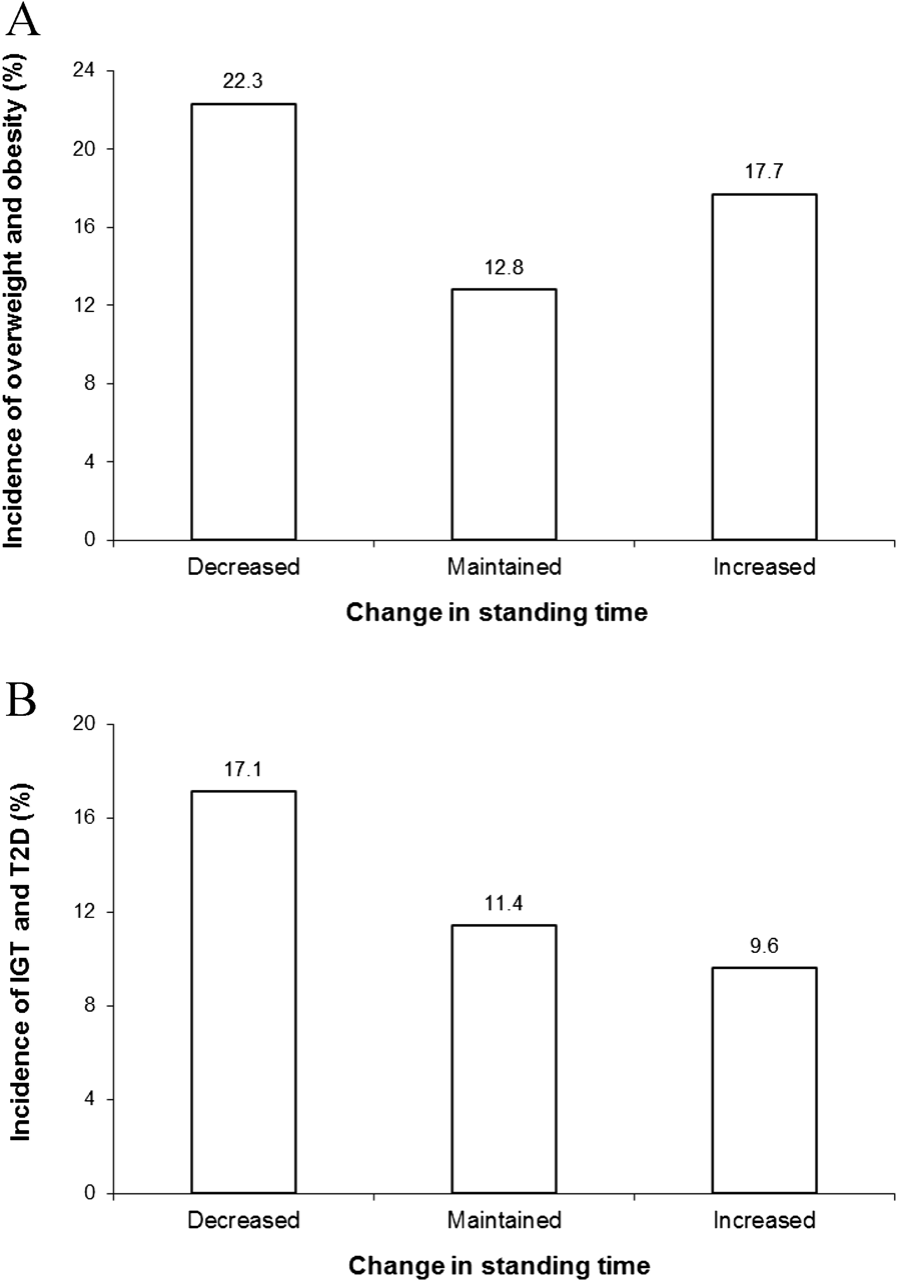
**Development of overweight/obesity (A) and impaired glucose tolerance/type 2 diabetes (IGT/T2D) (B) according to changes in standing time over the 6-year follow-up period in adults.** Data are presented as percentage. Overweight/obesity was defined as a body mass index ≥25 kg/m^2^. Impaired glucose tolerance/type 2 diabetes was defined according to the American Diabetes Association and the World Health Organization criteria [26,27]. Statistical significance was assessed by a chi-squared test (*P* < 0.05 for all analyses). Change in standing time categories: decreased (*n* = 82), maintained (*n* = 149) and increased (*n* = 62).

Finally, the incidence of OW/OB and IGT/T2D across categories of changes in standing time is shown in Figure 2. We observed that the change in standing time (from baseline to year 6) was significantly associated with the development of OW/OB and IGT/T2D, with higher incidence rates in adults reporting a reduction in standing time at follow-up. However, the associations became non-significant after adjustment for covariates, especially total annual family income and submaximal working capacity (data not shown).

## Discussion

Overall, we observed that greater amounts of occupational standing time are associated with a lower incidence of OW/OB and IGT/T2D in this sample of adults. However, greater time spent standing was not sufficient in and of itself to prevent the development of outcome measures. The addition of confounding factors to the models, especially annual income and cardiorespiratory fitness, led to non-significant associations. Thus, our results suggest that workplace standing time alone is not sufficient to prevent the incidence of OW/OB and IGT/T2D. Future efforts are needed to better understand the added value of standing time for overall health.

Sitting has become pervasive in today’s environment [30-32] and studies have provided compelling evidence that excessive sitting is associated with the development of several chronic diseases and premature mortality [2,5,19]. Recent intervention studies suggest that replacing sitting with standing may result in rapid and positive changes in important health markers [20,21,33]. For example, Buckley *et al.* compared the impact of an afternoon of seated vs. standing office work in a group of 10 adults [20]. In comparison to the seated condition, they reported that participants burned 174 kcal more during the standing condition, and also demonstrated a 43% lower glycemic response following a test meal. Similarly, Thorp *et al.* reported that alternating between sitting and standing every 30 minutes resulted in an 11% lower glycemic response to a test meal, when compared to a typical (*e.g.* sitting) workstation [21]. These intervention studies are supported by the recent paper of Katzmarzyk, who found that time spent standing was inversely associated with mortality risk [19]. Although standing appears to be a better choice than sitting for cardio-metabolic health, results of the present study suggest that other important factors should be considered (*e.g.* annual income and cardiorespiratory fitness) to ultimately prevent the development of chronic diseases.

The definition of standing time in the present study (*i.e.* time spent standing at work or during the main occupation) suggests that participants are probably not only standing still; light-intensity physical activity may also be involved in this amount of time spent standing. There is a growing body of evidence to suggest that light-intensity physical activity (*e.g.* walking) confers health benefits [16,34,35]. Although light-intensity physical activity is not typically part of physical activity guidelines, important health benefits can certainly be obtained at the lower end of the energy expenditure continuum [8].

There are a number of biological mechanisms through which greater time spent standing may lead to a reduced risk of chronic diseases. Standing may have a measurable impact on important metabolic processes, especially those related to glycemic control and lipoprotein metabolism. In comparison to a day of prolonged sitting, Latouche *et al.* showed that a day which incorporates light intensity activity breaks results in increased expression of genes related to lipid and carbohydrate metabolism in skeletal muscle [36]. Similarly, Hamilton *et al.* reported that standing results in large increases in lipoprotein lipase activity when compared to prolonged sedentary behavior in animal models [37]. In addition to any direct metabolic impact, standing may also be associated with reduced weight gain by slightly but consistently increasing energy expenditure, as suggested by the results of Buckley *et al.* [20].

There are several strengths and limitations of this study that warrant discussion. Strengths of this study include its longitudinal design and the adjustment for important variables that could confound the relationship between standing time and the incidence of chronic diseases (*e.g.* caloric intake, cardiorespiratory fitness and socioeconomic status). Furthermore, data were obtained from both men and women and we used an approach that should minimize confounding with repeated measures (baseline and year 6). However, our results need to be interpreted in light of the following limitations. First, the relatively small sample size limits statistical power and more high quality and well powered studies are needed to better appreciate the influence of standing time on the development of chronic diseases. The present study should thus be seen as an exploratory analysis which needs to be replicated in larger cohorts. Second, the exposure variable (standing time) was self-reported from a questionnaire and its validity is uncertain. Our findings are also limited to occupational standing, as opposed to total standing time. Third, the level of movement/activity while standing was not known and some people may have been more active than others during this period. Fourth, the external generalizability of our findings may be restricted to adults of Western European descent. Finally, the possibility of residual confounding by unmeasured variables is always a possibility in observational studies.

## Conclusions

In summary, the present study is the first to examine the association between occupational standing time and the incidence of OW/OB and IGT/T2D in adults. Our results suggest that standing time alone is not sufficient to prevent the incidence of OW/OB and IGT/T2D, which has implication for the risk of other chronic diseases. Replication studies with longer follow-up periods and larger sample sizes are needed to better understand the potential benefits of higher amounts of standing time throughout the day on the prevention of chronic diseases.
